# Determining effective drug concentrations for selection and counterselection genetics in *Drosophila melanogaster*

**DOI:** 10.1016/j.xpro.2021.100783

**Published:** 2021-09-14

**Authors:** Nick Matinyan, Yezabel Gonzalez, Herman A. Dierick, Koen J.T. Venken

**Affiliations:** 1 Verna and Marrs McLean Department of Biochemistry and Molecular Biology, Baylor College of Medicine, Houston, TX 77030, USA; 2 Integrative Molecular Biomedical Sciences Graduate Program, Baylor College of Medicine, Houston, TX 77030, USA; 3 Department of Molecular and Human Genetics, Baylor College of Medicine, Houston, TX 77030, USA; 4 Department of Neuroscience, Baylor College of Medicine, Houston, TX 77030, USA; 5 Department of Pharmacology and Chemical Biology, Baylor College of Medicine, Houston, TX 77030, USA; 6 Dan L. Duncan Comprehensive Cancer Center, Baylor College of Medicine, Houston, TX 77030, USA; 7 McNair Medical Institute at The Robert and Janice McNair Foundation, Baylor College of Medicine, Houston, TX 77030, USA

**Keywords:** Genetics, Model organisms

## Abstract

We recently integrated into fly genetics a set of four selection and two counterselection markers and their corresponding drugs that can be used individually or in combination. These markers eliminate the need to visually screen progeny. Before using these markers in new genetic backgrounds, effective selection/counterselection concentrations should be established for each marker/drug combination. This protocol describes how to set up, perform, and analyze a drug titration curve to determine the effective selection/counterselection drug concentrations for their corresponding markers.

For complete details on the use and execution of this protocol, please refer to Matinyan et al., 2021.

## Highlights

• Selection/counterselection in *Drosophila melanogaster*
• Establish a drug titration curve for a selection/counterselection marker
• Identify the optimal drug concentration for a selection/counterselection marker

## Graphical abstract

**Figure:**
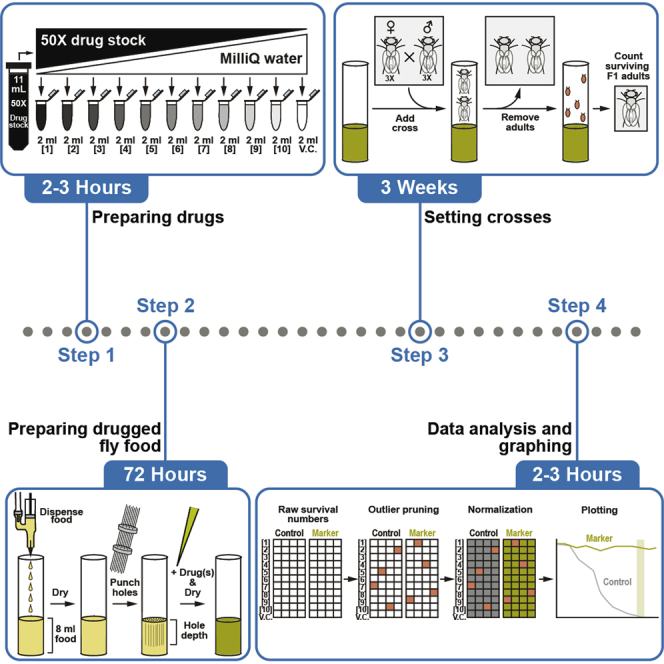

## Teaser

We recently integrated a set of four selection and two counterselection markers and their corresponding drugs into fly genetics that can be used individually or in combination. These markers eliminate the need to visually screen progeny. Before using these markers in new genetic backgrounds, effective selection/counterselection concentrations should be established for each marker/drug combination. This protocol describes how to set up, perform, and analyze a drug titration curve to determine the effective selection/counterselection drug concentrations for their corresponding markers.

## Before you begin

In a recent report, we developed a drug-based, selection and counterselection platform for multiplexed genetic manipulation in *Drosophila melanogaster* (Matinyan et al., 2021). We designed a compact expression cassette to test four different drug resistance and two drug sensitivity markers. We successfully selected resistant transgenic animals using the drugs G418 sulfate, puromycin, blasticidin S, and hygromycin B. We also demonstrated effective, drug-based counterselection against sensitized animals using either ganciclovir or 5-fluorocytosine. We then applied the selection/counterselection platform to make double transgenic animals in a single step, generated selectable and counterselectable balancer chromosomes, and made fluorescently tagged selectable *P[acman]* BAC transgenics (Matinyan et al., 2021). To facilitate expanding the platform to new applications, using it in different genetic backgrounds, or developing new markers that are resistant or sensitive to novel drugs, we provide a detailed protocol to perform drug titration curves to identify the optimal concentration for selection or counterselection.

1. Expand the fly stocks you will be testing.

***Note:*** A typical titration curve featuring vehicle control and ten drug concentration testing points consists of four replicate vials per drug condition each containing three female and three male flies, totaling 264 flies (132 female and 132 male). These numbers can be adjusted when designing a smaller curve or if using more flies per vial (We recommend at least 2 of each sex per vial).

***Note:*** In this protocol we demonstrate both a full eleven point titration curve and a second, smaller one. A smaller curve centered around the previously described effective selection or counterselection concentration for a given maker/drug combination (Matinyan et al., 2021) can also be carried out instead.

2. Order the EGFP control strain as well as the relevant drug marker expressing strains that were previously described (Matinyan et al., 2021).

***Note:*** The EGFP control strain and the relevant drug marker expressing strain will serve as negative and positive controls, respectively, while performing drug titration experiments to establish an effective selection concentration (ESC) or effective counterselection concetration (ECC) in the desired genetic background.

***Note:*** Stocks can be ordered from the Bloomington Drosophila Stock Center (https://bdsc.indiana.edu/).

***Note:*** See Table 1 for a full list of markers and Table 2 for a full list of relevant fly stocks.

***Note:*** For best results we recommend using younger flies no older than 3 weeks. Older flies tend to produce fewer offspring, which may lead to more variation between vials per point making results more difficult to interpret.

***Note:*** We perform all our selection/counterselection experiments in a 25°C incubator (12 h light-dark cycle, ambient humidity) to speed up larval development. However, the drug titration can be carried out between 18°C and 25°C, though temperatures lower than 25°C will prolong the length of the experiment.

***Note:*** Exposure to selection/counterselection agents slows down larval development by about a week, which can be somewhat offset by elevated temperature during development.

**Table 1:** Summary of selection/counterselection markers and respective drugs

Marker	Marker type	Encoded protein	Marker size (bp)	Drug(s)	Solvent	Cost per vial ($/vial)^b^
***nptII***	Selection/Drug resistance	Neomycin phosphotransferase II	795	G418 sulfate (Geneticin)	MQ H2O	0.07
***pac***	Selection/Drug resistance	Puromycin N-acetyltransferase	600	Puromycin HCl	MQ H2O	2.32–4.64
***bsr***	Selection/Drug resistance	Blasticidin S-resistance	423	Blasticidin S	MQ H2O	0.41–0.73
***hgh***	Selection/Drug resistance	Hygromycin B phosphotransferase	1026	Hygromycin B	MQ H2O	0.04–0.05
***sr39TK***	Counterselection/Drug sensitivity	Thymidine kinase	1131	Ganciclovir	0.1N NaOH	0.01–0.04
***FCU1***	Counterselection/Drug sensitivity	FCU1	1122	5-Fluorocytosine	1**×** PBS^a^	<0.01

List of six orthogonal selection/counterselection markers that either confer drug resistance or sensitivity to marker expressing fly stocks. Markers and their corresponding drugs can be used in conjunction for complex, multiplexed genetic manipulations without compromising the robustness of the individual drugs at selecting/counterselecting the relevant animals.

a Requires rigorous mixing to fully dissolve in PBS.

b Cost per vial indicated is at the effective selection and counterselection concentrations determined in the primary research paper (Matinyan et al., 2021).

**Table 2:** Summary of control marker expressing fly strains

Fly strain genotype	Abbreviated name	Description	BDSC #
*y[1] w[1118]; PBac{y[+mDint2] w[+mC]=Hsp70-CP6-EGFP}VK00033*	EGFP	EGFP expressing control strain	92331
*y[1] w[1118]; PBac{y[+mDint2] w[+mC]=Hsp70-CP6-G418R}VK00033*	G418^R^	G418 resistant stock	92332
*y[1] w[1118]; PBac{y[+mDint2] w[+mC]=Hsp70-CP6-PuroR}VK00033*	Puro^R^	Puromycin resistant stock	92333
*y[1] w[1118]; PBac{y[+mDint2] w[+mC]=Hsp70-CP6-BlastR}VK00033/TM6B, Tb[1]*	Blast^R^	Blasticidin resistant stock	92334
*y[1] w[1118]; PBac{y[+mDint2] w[+mC]=Hsp70-CP6-HygroR}VK00033*	Hygro^R^	Hygromycin resistant stock	92335
*y[1] w[1118]; PBac{y[+mDint2] w[+mC]=Hsp70-CP6-GCVS}VK00033*	GCV^S^	Ganciclovir and acyclovir sensitive stock	92337
*y[1] w[1118]; PBac{y[+mDint2] w[+mC]=Hsp70-CP6-5FCS}VK00033*	5FC^S^	5-Fluorocytosine sensitive stock	92338

List of marker expressing strains to be used as either negative control (EGFP) or as positive controls (G418^R^, Puro^R^, Blast^R^, Hygro^R^, GCV^S^, 5FC^S^) during drug titrations curves. All lines are available from the Bloomington Drosophila Stock Center (https://bdsc.indiana.edu/).

## Key resources table

**Table:** 

REAGENT or RESOURCE	SOURCE	IDENTIFIER
**Chemicals, peptides, and recombinant proteins**		

G418 sulfate	VWR	Cat# 97063-060
Puromycin dihydrochloride	VWR	Cat# 97064-280
Blasticidin S hydrochloride	VWR	Cat# 71002-676
Hygromycin B	VWR	Cat# AAJ6068103
Ganciclovir	TCI America	Cat# G0315
5-Fluorocytosine	TCI America	Cat# F0321
Nutri-Fly Drosophila Agar	Genesee Scientific	Cat# 66-103
Active dry yeast	Red Star®	Cat# 15700
Cornmeal	VWR	Cat# 75860-346
Dextrose monohydrate	VWR	Cat# JT1910-5
D-(+)-Sucrose	VWR	Cat# BDH9308
Tegosept	Genesee Scientific	Cat# 20-258
Propionic Acid	Sigma-Aldrich	Cat# P1386

**Experimental models: Organisms/strains**		

Drosophila stock: y[1] w[1118]; PBac{y[+mDint2] w[+mC]=Hsp70-CP6-EGFP}VK00033	PI: Koen JT Venken Citation: Matinyan et al., 2021	BDSC Cat# 92331
Drosophila stock: y[1] w[1118]; PBac{y[+mDint2] w[+mC]=Hsp70-CP6-G418R}VK00033	PI: Koen JT Venken Citation: Matinyan et al., 2021	BDSC Cat# 92332
Drosophila stock: User’s genetic background/strain of choice	User provided	n/a

**Software and algorithms**		

Prism v9	Graphpad	https://www.graphpad.com/scientific-software/prism/

**Other**		

Drosophila Vial, Narrow	VWR	Cat# 75813-158
Drosophila Vial Plugs, Cellulose Acetate	VWR	Cat# 89168-886
15 mL conical centrifuge tubes	Nunc	Cat# 89174-468
Piston-driven pump	Filamatic	Cat# DAB-8-4
5 mL snap-cap tubes	Eppendorf	Cat# 0030119380
XPE504 Analytic Balance	VWR	Cat# 10025-668
Microspatula	VWR	Cat# 80071-668
0.5 mm metal rod, 8–10’’ in length	n/a	n/a
Kitchen cutting board	n/a	n/a
Flystuff Ultimate Flypad	Genesee Scientific	Cat# 59-172
Benchtop Flowbuddy	Genesee Scientific	Cat# 59–122B
Flystuff Blowgun	Genesee Scientific	Cat# 54-104
Stereo dissecting microscope	n/a	n/a
Drosophila incubator	SHEL Lab	Cat# SRI20PF
Rubber Tubing	Genesee Scientific	Cat# 59-125
CO_2_	Airgas	Cat# CD 50
Fine point brush	n/a	n/a

## Materials and equipment

***Alternatives:*** This protocol uses a large electric kitchen kettle to prepare fly food (up to 30 liters). Alternatively, for smaller amounts of fly food, a Crockpot that can be purchased from any department store can be used.

**Figure 1:**
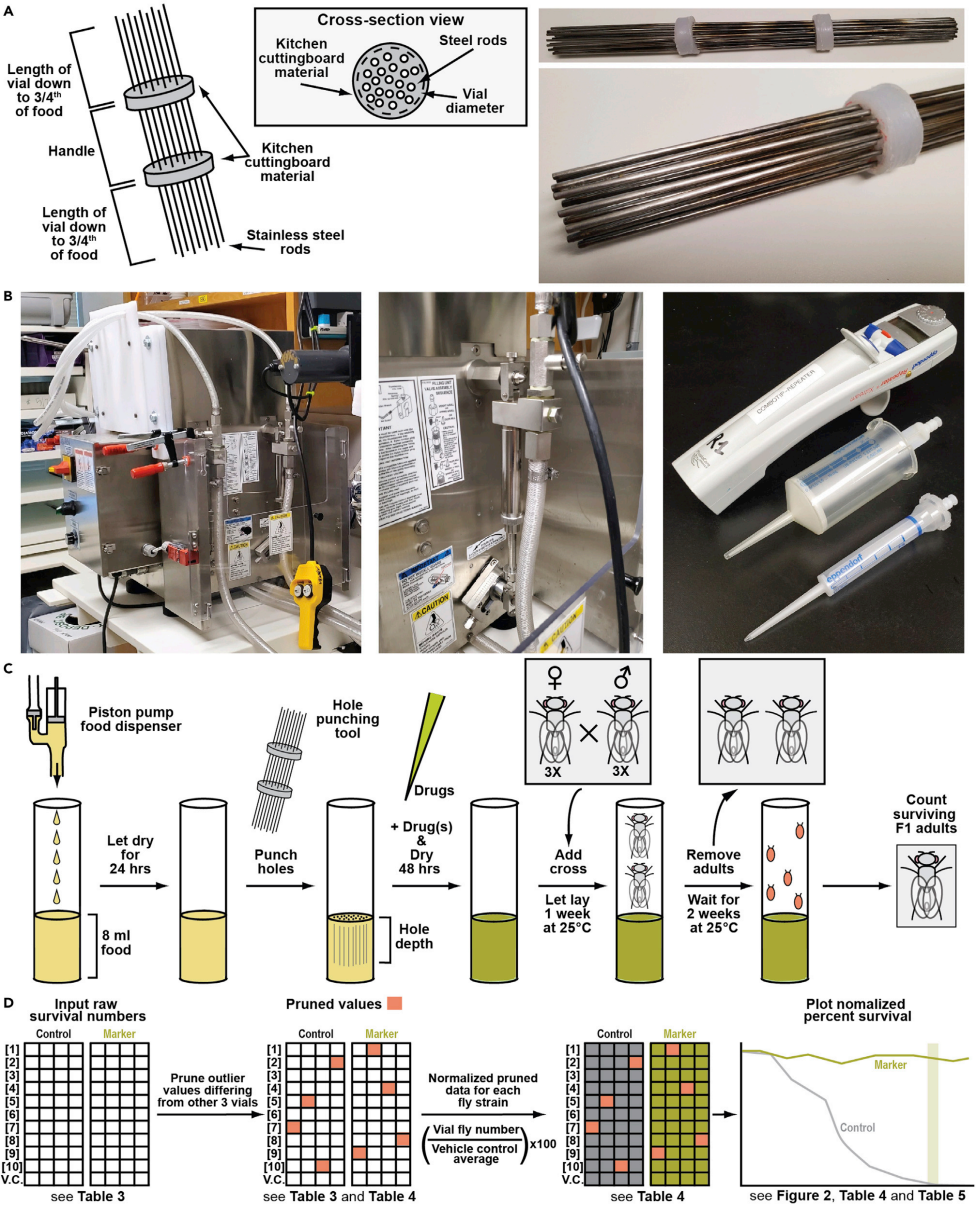
Drug titration experimental setup and workflow (A) Design and construction of a homemade hole punching tool. The tool is made up of twenty 12” stainless-steel rods hot glued into two plastic discs made of kitchen cutting board material. Holes for the rods were made using a battery-operated electric drill (MATRIX™ Quick Connect System from Black & Decker) and rods positioned in place before hot glueing by heating up the rods using a torch lighter. The discs were measured to be slightly bigger than the diameter of a standard fly vial and positioned in such a way that the rods reach only three-fourths of the way down into the food as a compromise between drug percolation and structural integrity of the food plug. The exact dimensions of the discs will depend on the type of vials used (i.e., wide versus narrow). (B) Pictures of a quadruple piston pump (left), zoom in of a single piston pump unit (middle), and multipipette repeater with 50 mL and 5 mL combitips used for precise food or drug dispensing, respectively. (C) Drugged food is prepared by first precisely and accurately dispensing 8 mL of fly food into a vial using a piston pump food dispenser (see B). Alternatively, food can be hand dispensed using a multipipette repeater and accompanying 50 mL combitip (see B). Once food is dispensed, it is allowed to air dry for at least 12 h covered by cheescloth before being hole punched using the homemade hole punching tool. Drug (see Table 1) is then dispensed using a multipipette repeater and accompanying 5 mL combitip (see B), and allowed to percolate as the vials dry another 48 h covered by cheesecloth, followed by plugging each vial. Adult flies, three of each sex per strain, are then added to vials and allowed to lay eggs for 1 week at 25°C before being removed. Larvae are then allowed to develop for an additional two weeks. Resulting adults, if any, are then counted and the data reported as normalized percent survival (see D). (D) Simplified schematic of data analysis for resulting fly survival numbers from a drug titration experiment. Briefly, raw surviving fly numbers are tabulated (see Table 3), and obvious outlier values which disagree with the other 3 biological replicates are pruned (orange) (see Tables 3 and 4). For each strain a mean fly survival number is calculated for vehicle control treated vials. All values for a given strain are then normalized to the vehicle control by dividing by this mean (see Table 4). The effective selection concentration (ESC) or effective counterselection concentration (ECC) is determined after a 2-way ANOVA with multiple comparisons analysis (see Table 5). Normalized values are then plotted with the ESC indicated in green (see example in Figure 2C), or the ECC indicated in purple (see example in Figure 2D). See the quantification and statistical analysis section for further details.

***Alternatives:*** This protocol uses a piston-driven pump to precisely and accurately dispense fly food (Figure 1B, **left**). However, an alternative is to use a Multipipette Repeater E3 (Eppendorf, 4987000118) alongside Combitips®, 50 mL (Eppendorf, 0030089596) (Figure 1B, **right**) to dispense food. Alternatively, if neither is available, we would recommend manually ladling food into vials, marked with a stripe indicating the height of the required volume of food, in a precise manner to ensure accurate fly food amount in all the vials.

***Alternatives:*** This protocol uses small stainless steel rods (0.5 mm diameter and 8–12’’ in length) to poke holes in the fly food vials prior to adding the drugs. As an alternative we have built a custom tool for faster and more reproducible hole punching of food vials using kitchen cutting board material and 20 12” stainless steel rods (Figure 1**A**). Assembly of the tool is relatively simple and expedites hole punching the food.

***Alternatives:*** This protocol describes addition of drug to dry fly food vials using standard pipette tips and pippetters. An alternative is to use a Multipipette Repeater E3 (Eppendorf, 4987000118) alongside Combitips®, 5 mL (Eppendorf, 0030089456) to speed up drug dispensing.

**Table:** Fly food recipe

Reagent (storage)	Amount	Final concentration (w/v)
Nutri-Fly Drosophila agar (RT)	6.4 g	0.64
Active dry yeast (RT)	30 g	3
Dextrose monohydrate (RT)	55 g	5.5
D-(+)-Sucrose (RT)	30 g	3
Cornmeal (RT)	70 g	7
Tegosept, 20%, in ethanol (RT)	4 mL	0.4
Propionic acid (RT)	4 mL	0.4
Water (Tap, RT)	Up to 1 L	80.06
**Total**	**1 L**	**100%**

Store at RT or 4°C for longer storage up to a month.

## Step-by-step method details

### Preparing fly food

**Timing: 2 h active time; 24 h total**

1. Add appropriate amount of water to kettle/Crockpot and begin heating at medium-high heat bringing to a boil/simmer.

***Note:*** If using a Crockpot add appropriate amount of water and set to high heat, making sure to close the lid. Typically, 1 liter of food yields about 120 vials, each containing 8 mL.

**CRITICAL:** If using a standing mixing element, set it to a medium-high speed and continuously mix the solution. Otherwise, occasionally mix the food by hand every 10–15 min to ensure the dry ingredients do not stick to the bottom and burn.

2. Mix all dry ingredients together in a large bowl according to the following recipe: 6.4 g agar, 30 g dry active yeast, 70 g cornmeal, 55 g dextrose and 30 g sucrose per liter of water.
3. Make sure the dry ingredients are well mixed with no major clumps.

***Note:*** Dextrose and sucrose, depending on ambient humidity, are prone to forming large clumps which must be broken up completely before addition to the water.

4. Slowly sift mixed dry ingredients into the kettle using a large scoop or weigh boat.
5. Maintain mixing speed when using a mixing element.

***Note:*** When no mixing element is available, stir manually after each addition of dry ingredients, ensuring they are fully dissolved before adding more.

***Note:*** Slow addition of dry ingredients ensures even mixing throughout the solution resulting in a smooth texture of the final food product.

6. Cook food until agar is fully dissolved.

***Note:*** Depending on the cooking pot that is used, this may take anywhere from 45 min (electric kettle) to 3 h (Crockpot).

***Note:*** Once the food has reached ∼70°C the agar should be fully dissolved. The resulting product should resemble a thin polenta and have a slightly sweet smell.

7. Stir throughout the cooking time.

***Note:*** The user may choose to follow their own cooking style, which will likely yield similar results as long as drugs are added to cool, solidified food after it has been dispensed into individual vials.

8. Once food is cooked, reduce heat allowing the food to cool down to 70°C.
9. Reduce stirring speed or stir manually on occasion.

***Note:*** If using a Crockpot, the food will likely not be much hotter than 70°C.

***Optional:*** To speed up cooling, initial water volume can be reduced, though not the dry components (*e.g.,* 8 liters but ingredients for 10 liters). Missing volume can be added as tap water containing ice by slowly sifting in the appropriate volume using a large scoop to avoid excessive bubbling.

10. Once food has cooled to 70°C, slowly add 20% Tegosept (4 mL per liter) and propionic acid (4 mL per liter).

***Note:*** Care should be taken in addition to avoid bubbling.

11. Maintain stir speed for 5 min.
12. Reduce heat and stir speed making sure to keep the food liquid.

13. Accurately and precisely dispense 8 mL of liquid food into vials using piston pump or alternative method (Figure 1C).
  ***Alternatives:*** If available pump is not accurate or precise enough, food can be dispensed manually using a Multipipette Repeater.
  a. Take a 50 mL Combitip. Using wire cutters or similar, cut the tip of the Combitip off (about 1–2 cm). It may also be possible to dispense food using a catheter tip syringe.
  b. Program the repeater to dispense 8 mL of food 6 times.
  c. Dispense using cut Combitip.
    ***Note:*** If Combitip becomes clogged, eject any remaining liquid, eject the tip, manually pump the tip to remove the clog. If still clogged, replace with new cut tip as above.
    ***Alternatives:*** If neither accurate pump nor repeater are available an alternative is to manually ladle fly food into each vial, marked with a stripe indicating the required volume to make sure to add exactly 8 mL.

14. Once food has been dispensed, cover with cheesecloth or similar and leave to dry unplugged (without plugs) for at least 12 h.

**Figure 2:**
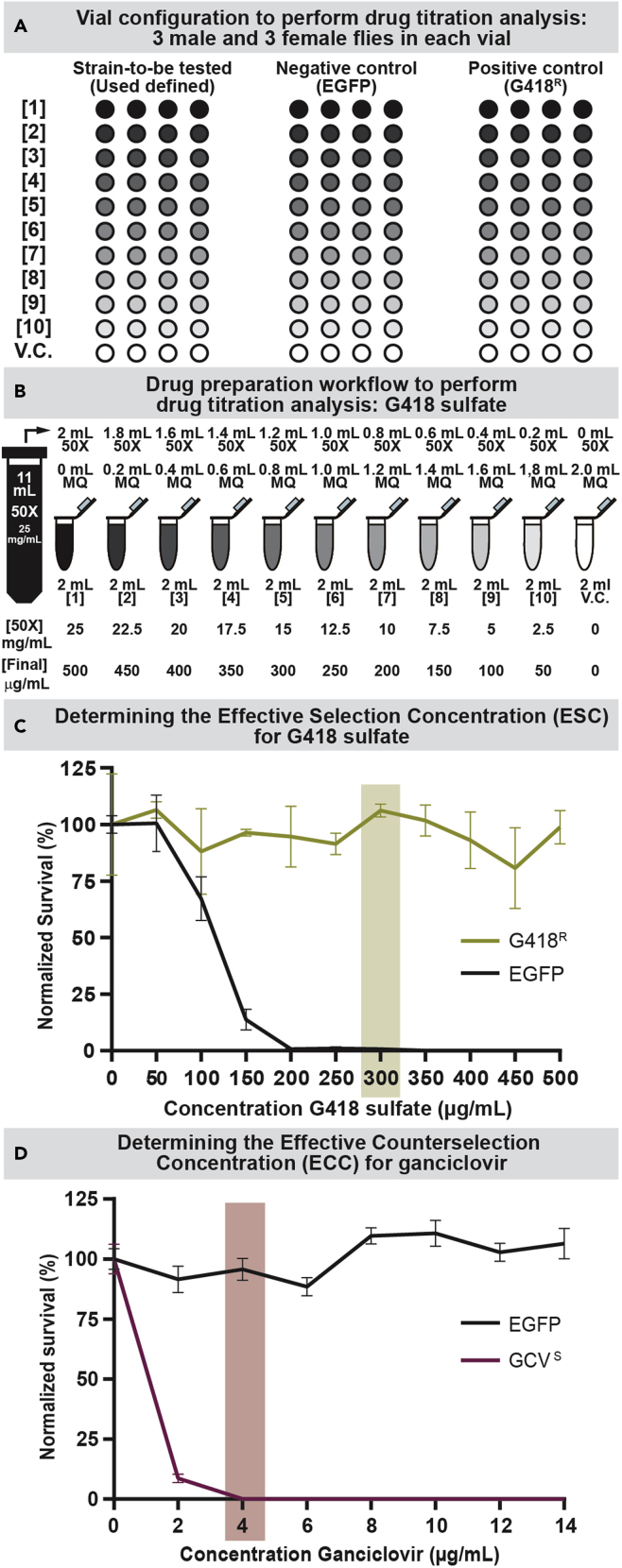
Example survival curves determining effective selection or counterselection drug concentrations (A) Vial configuration to perform drug titration analysis testing a user specified strain against a negative control strain (EGFP) and a positive control strain (G418^R^). An 11-point (including vehicle control) titration curve with a single experimental strain (user’s choice), a negative control strain (EGFP control strain) (see Table 2), and a positive control strain (marker expressing strain) requires a minimum of 132 fly food vials (44 per strain, 4 per drug concentration). (B) Drug preparation workflow to perform drug titration analysis. Eleven mL of 50**×** master stock is made and diluted with MQ water over eleven 2 mL tubes, as indicated, resulting in the final concentrations and working dilutions, as indicated. (C) Example survival data showing a titration curve for the drug G418 sulfate. A G418 sulfate resistant strain (G418^R^) was compared to the EGFP control strain (Control). The concentration highlighted in green represents the effective selection concentration (ESC) at which only the resistant animals survive while all control animals are eliminated without significantly affecting G418 resistant strain survival versus vehicle control. Error bars represent S.E.M. for at least 3 biological replicates per condition per strain. (D) Survival data showing a titration curve for the counterselection agent ganciclovir. A sensitized strain (GCV^S^) was compared to the control strain (EGFP). The concentration highlighted in purple represents the effective counterselection concentration (ECC) at which only the control strain survives while all sensitized animals are eliminated without significantly affecting control strain survival versus vehicle control. Error bars represent S.E.M. for at least 3 biological replicates per condition per strain.

***Note:*** A 10-point (plus vehicle control) titration curve with a single experimental strain (user’s choice), a negative control strain (EGFP control strain) (Table 2), and a positive control strain (marker expressing strain) requires a minimum of 132 fly food vials (44 per strain; 4 per drug concentration) (Figure 2A).

***Note:*** The EGFP control strain features the EGFP fluorescent marker expressed using the same expression cassette as the drug resistance/sensitivity markers. It was inserted into the same genomic location (VK00033) and in the same genetic background as the positive marker control strains to eliminate any position effect differences when comparing different marker strains (Matinyan et al., 2021).

***Note:*** The exact number required will depend on the user’s experimental design, though we recommend following our standard setup at least once before varying conditions. The example provided in this protocol uses 132 vials as described below. Troubleshooting 1.

### Drugging prepared food

**Timing: 2 h active, 48 h total**

***Note:*** While this protocol describes setting up a titration curve for G418 sulfate, it is applicable for any of the other selection (puromucin, blasticidin S, or hygromycin B) or counterselection (ganciclovir or 5-fluorocytosine) drugs previously described (Matinyan et al., 2021), or any novel to-be-tested drug. The amount of each drug, solvent, and relevant positive control varies depending on drug. See Table 1 for a full list of drugs, solvents, *etc*.

15. Make a 50**×** solution of drug for the highest working concentration to be used (Figure 2B).
  ***Note:*** In this example the highest concentraton will be 25 mg/mL. Addition of 160 μL of drug to each 8 mL food vial represents a dilution factor of 50, hence the 50× master stock. We describe setting up a 10–point titration curve (and an 11^th^ point for the vehicle control, MQ H_2_O) from 2.5 mg/mL to 25 mg/mL of G418 sulfate. The resulting concentrations in the food vials will range from 50 to 500 μg/mL with a 50 μg/mL step size.
  ***Note:*** When calculating the amount of drug needed it is important to consider the volume of drug required to adequately treat all vials. For one food vial, containing 8 mL of food, 160 μL of drug is needed. In this example, we will be making 12 vials per drug concentration (4 vials per concentration for each of 3 fly strains). The minimum volume required will be 1.92 mL of drug (160 μL multiplied by 12). However, it is recommended to always make slightly more drug than is minimally required because there will likely be some pipetting error. We will be making 2 mL of drug per dilution to ensure all vials receive the right amount of drug.
  a. Measure out 275 mg of G418 sulfate into the 15 mL conical using the analytic balance and microspatula.
  b. Dissolve in 11 mL of MQ H_2_O by inverting conical 5–10 times resulting in a solution with concentration 25 mg/mL.
    ***Note:*** More rigorous mixing may be necessary depending on drug used requiring use of a vortexer. See Table 1 for solvent details.

16. Make a dilution series by adding the appropriate amount of 50**×** stock solution to each of the nine 5 mL tubes.

***Note:*** We recommend making the stock solution fresh each time, but any remainder can be stored for up to 1–2 months at −20°C.

17. Add MQ H_2_O to dilute drug to appropriate concentration resulting in a dilution series each with a final volume of 2 mL.
18. Poke several holes, 8–10, about 3/4^th^ of the way into each of the 132 vials from the previous step.

***Note:*** Hole poking will allow added drug to percolate more fully and evenly into the food (Figure 1C).

19. Add 160 μL of each drug concentration to hole punched vials using a standard pipette and tips or repeater (Figure 1C).

***Note:*** For each concentration, we make four biological replicates (four vials) per fly strain. This results in 44 vials per fly strain (including 4 vials treated with vehicle control) and 132 total vials for an experiment involving three strains (Positive control, negative control, experimental).

20. Let drugged food stand unplugged, but covered with cheesecloth or similar, to avoid contamination, for 48 h allowing drug to fully soak into the hole-punched food.
21. Plug vials.

### Setting up the flies

**Timing: 2–3 h**

22. Collect the appropriate number of flies for each strain and separate them by sex.
  ***Note:*** We recommend using flies no older than 3 weeks of age as fecundity begins to decrease in older flies and may increase variability between repeats.
  a. Anesthetize flies of each strain using blowgun and dump onto flypad with CO_2_ running.
  b. Separate into male and female (females do not have to be virginal) using the brush under the dissecting scope.
  c. Add separated flies to fresh vials containing regular food.
    ***Note:*** Avoid crowding flies. Limit number of flies per vial to ∼60 animals.
    **Recommended:** While flies are recovering from CO_2_, tip vial on its side to prevent animals from falling into food and becoming stuck.
  d. Once flies have recovered, place into 25°C incubator for at least 1 h.
    ***Note:*** We recommend separating the flies the day before setting up the experiment, while the drugged food is still drying, and allowing them to recover at 25°C for at least 12 h for best results.

23. Anesthetize separated flies of each sex for a given strain and place on flypad with CO_2_ running.

***Note:*** We suggest doing this one vial at a time per gender per strain to minimize the amount of time spent by the flies under CO_2_.

24. To each vial for a given strain, add three males and three females (Figure 1C).
  a. Use the brush to sweep flies about halfway into the sideways vial.
  b. Plug the vial and leave sideways while flies recover.
  c. Repeat for each vial per concentration for each strain.
  d. Once flies have fully recovered from CO_2_ place the vials upright in an appropriately sized tray.

### Running and analyzing the drug titration curve

**Timing: 3 weeks**

25. Place drugged vials loaded with flies as described in the previous step into the incubator set to 25°C.
26. Allow flies to mate/lay eggs for 1 week.
27. Remove all surviving adult flies by inverting the vials and tapping adults into an ethanol trap.
28. Allow eggs/larvae to develop for an additional 2 weeks in the incubator at 25°C.

***Note:*** Flies should begin eclosing at the start of the second week. Drug treatment slows development of larvae by approximately 1 week versus vehicle control. By the end of the 3^rd^ week all surviving flies should have eclosed. Non-resistant animals treated with selection drug tend to die during 1^st^–2^nd^ larval instars. Conversely, drug sensitized animals exposed to counterselection drugs die early in pupation.

29. Remove vials from incubator.

30. Anesthetize flies one vial at a time.
  a. Dump flies onto flypad with CO_2_ flowing.
  b. Count the number of adult flies taking care to include any adults that may have eclosed but fallen into the food.
    ***Note:*** Although the sex of the surviving animals can be noted, we have not observed any sex bias drug resistance for any of the marker/drug combinations.
  c. After recording the count, dump flies into waste container, such as an ethanol trap.
  d. Repeat until all vials are counted. Troubleshooting 2.

31. Normalize survival data by averaging the four vehicle control replicates for a given strain and then dividing all fly counts for that strain by this number (Figure 1D).

***Note:*** We report survival data as normalized percent survival versus vehicle control. Data are presented as the average percent survival for a given drug concentration for each strain tested with error bars representing standard error of the mean. We provide example curves for determining either an effective selection concentration for G418 sulfate (Figure 2C) or an effective counterselection concentration for ganciclovir (Figure 2D).

***Note:*** We advise four biological replicates for each concentration per strain to ensure enough replicates per point for statistical analysis. Sometimes a cross fails due to infertility or other factors producing little to no progeny and is obviously different from the other three vials for that drug concentration. Four replicates ensures that, in most cases, there will still be at least three replicates per data point. Obvious outliers should be excluded from averages and future analysis. Troubleshooting 3.

***Note:*** We use the statistical analysis and graphing software Prism 9 (Graphpad) to analyze and visualize our data. However, the user may use any similar software for their analysis.

## Expected outcomes

A successful drug titration experiment should yield a curve clearly showing the effective selection concentration (ESC) (Figure 2C), or effective counterselection concentration (ECC) (Figure 2D). We define the ESC as the minimal drug concentration at which the control (non-resistant) fly strain shows no viability while the corresponding marker expressing strain (resistant) is unaffected. Conversely, we define the ECC as the minimal drug concentration at which the sensitivity marker expressing strain is totally eliminated while the control strain (not sensitive) is unaffected. We recommend setting up at least 4 vials per concentration per strain per drug being tested to ensure that there will be at least three vials with fertile crosses allowing for robust statistical analysis (See quantification and statistical analysis section below). A typical experiment should yield values like the provided example values shown in Table 3.

**Table 3:** Surviving fly number counts

Concentration (μg/mL)	EGFP/Control vial 1	EGFP/Control vial 2	EGFP/Control vial 3	EGFP/Control vial 4	G418^R^ vial 1	G418^R^ vial 2	G418^R^ vial 3	G418^R^ vial 4
0 (Vehicle control)	100	88	102	89	128	94	**27∗**	56
50	122	92	65	102	**26∗**	92	103	101
100	55	82	54	**18∗**	**0∗**	49	87	109
150	6	19	22	5	**0∗**	92	88	88
200	1	1	1	0	90	65	108	**0∗**
250	3	1	0	0	94	90	80	75
300	1	0	0	2	**0∗**	94	98	103
350	0	0	0	0	95	83	105	**0∗**
400	0	0	0	0	79	100	57	109
450	0	0	0	0	80	**30∗**	79	110
500	0	0	0	0	73	106	94	93

Surviving fly number counts from a typical drug titration curve for G418 sulfate. Values marked in bold with an asterisk denote excluded values deemed to be outliers based on the values of the three other vials for that condition and strain. These outlying values are pruned from the dataset.

Once the ESC/ECC has been determined for a drug in a particular genetic background, drug-based selection and/or counterselection should be robust, with only resistant animals surviving treatment (Figure 2C) or sensitized animals being eliminated (Figure 2D). As all six of the markers are dominantly expressed, a single copy is enough to confer drug resistance/sensitivity. Therefore, when crossing two backgrounds with different ESC/ECC, progeny should be selected for or counterselected against using the higher ESC/ECC, respectively. Similarly, if crossing to a strain with an unknown ESC/ECC for a particular drug, we recommend using the known drug concentration, modifying it for future crosses if escapers are noted among the progeny.

## Quantification and statistical analysis

A successful drug titration curve should yield raw values like those shown in Table 3. For each vial, the total number of resulting flies after 3 weeks were counted and tabulated for each vial per concentration per strain. As mentioned above, on occasion a cross will not be fertile or will otherwise yield aberrant fecundity differing from the result of the other 3 vials. In these instances, we treat this value as an outlier (denoted in blue with an asterisk) and exclude it from further analysis. Troubleshooting 4. Row statistics are then performed on pruned data to determine the mean of each row per group (strain tested). For each strain all non-excluded values are then normalized to corresponding vehicle control mean fly number by dividing all values by the mean of the vehicle control vials and then multiplied by 100 to yield the normalized percent survival values (Table 4). The averaged values per concentration per strain are then graphed yielding survival curves as shown (Figures 2C and 2D). Finally, we perform a 2-way ANOVA with multiple comparisons using Dunnett’s multiple comparison test, comparing each row average per strain to the corresponding vehicle control row average (Table 5). This allows determination of the Effective selection/counterselection concentration (ESC/ECC) by ensuring that survival of the resistant (in the case of ESC) or the control (in the case of ECC) strain is not significantly different from vehicle control due to drug treatment at the putative ESC/ECC. Troubleshooting 5.

**Table 4:** Pruned normalized percent surviving fly number counts

Concentration (μg/mL)	EGFP/Control vial 1	EGFP/Control vial 2	EGFP/Control vial 3	EGFP/Control vial 4	G418^R^ vial 1	G418^R^ vial 2	G418^R^ vial 3	G418^R^ vial 4
0 (Vehicle control)	105.54	92.88	107.65	93.93	138.13	101.44		60.43
50	128.76	97.10	68.60	107.65		99.28	111.15	108.99
100	58.05	86.54	56.99			52.88	93.88	117.63
150	6.33	20.05	23.22	5.28		99.28	94.96	94.96
200	1.06	1.06	1.06	0.00	97.12	70.14	116.55	
250	3.17	1.06	0.00	0.00	101.44	97.12	86.33	80.93
300	1.06	0.00	0.00	2.11		101.44	105.76	111.15
350	0.00	0.00	0.00	0.00	102.52	89.57	113.31	
400	0.00	0.00	0.00	0.00	85.25	107.91	61.51	117.63
450	0.00	0.00	0.00	0.00	86.33		85.25	118.70
500	0.00	0.00	0.00	0.00	78.78	114.39	101.44	100.36

An example of pruned normalized percent survival values obtained from raw fly counts. For each strain, values were divided by the mean of the corresponding vehicle control vials and multiplied by 100. Empty cells represent excluded raw values.

**Table 5:** Example statistical determination of G418 sulfate effective selection concentration

EGFP control	Predicted (LS) mean diff.	95.00% CI of diff.	Below threshold?	Summary	Adjusted P value
**VC vs. 50 μg/mL**	−0.5277	−31.03 to 29.97	No	ns	>0.9999
**VC vs. 100 μg/mL**	32.81	−0.1364 to 65.75	No	ns	0.0515
**VC vs. 150 μg/mL**	86.28	55.78 to 116.8	Yes	∗∗∗∗	<0.0001
**VC vs. 200 μg/mL**	99.21	68.71 to 129.7	Yes	∗∗∗∗	<0.0001
**VC vs. 250 μg/mL**	98.94	68.45 to 129.4	Yes	∗∗∗∗	<0.0001
**VC vs. 300 μg/mL**	99.21	68.71 to 129.7	Yes	∗∗∗∗	<0.0001
**VC vs. 350 μg/mL**	**100**	**69.50 to 130.5**	**Yes**	**∗∗∗∗**	**<0.0001**
**VC vs. 400 μg/mL**	100	69.50 to 130.5	Yes	∗∗∗∗	<0.0001
**VC vs. 450 μg/mL**	100	69.50 to 130.5	Yes	∗∗∗∗	<0.0001
**VC vs. 500 μg/mL**	100	69.50 to 130.5	Yes	∗∗∗∗	<0.0001

G418^R^	Predicted (LS) mean diff.	95.00% CI of diff.	Below threshold?	Summary	Adjusted P value
**VC vs. 50 μg/mL**	−6.475	−41.53 to 28.58	No	ns	0.9992
**VC vs. 100 μg/mL**	11.87	−23.19 to 46.93	No	ns	0.9420
**VC vs. 150 μg/mL**	3.597	−31.46 to 38.66	No	ns	0.9996
**VC vs. 200 μg/mL**	5.396	−29.66 to 40.46	No	ns	0.9994
**VC vs. 250 μg/mL**	8.543	−24.25 to 41.34	No	ns	0.9893
**VC vs. 300 μg/mL**	−6.115	−41.17 to 28.94	No	ns	0.9993
**VC vs. 350 μg/mL**	−**1.799**	−**36.86 to 33.26**	**No**	**ns**	**0.9998**
**VC vs. 400 μg/mL**	6.924	−25.87 to 39.72	No	ns	0.9965
**VC vs. 450 μg/mL**	3.237	−31.82 to 38.30	No	ns	0.9996
**VC vs. 500 μg/mL**	1.259	−31.54 to 34.05	No	ns	0.9999

Results of a multiple comparison test using Dunnett’s multiple comparison method. The mean of each of the non-excluded replicates per concentration per strain were compared to the mean of each strain’s respective vehicle control vials to determine if survival at a given drug concentration was significantly different from control. The row in bold represents the empirically determined effective selection concentration (ECC) for EGFP strain flies (EGFP). At this concentration, no control strain flies (EGFP) survive to adulthood while the survival of drug resistant strain flies (G418^R^) is not significantly affected compared to its respective vehicle control treated vials. If there are any missing values (pruned outliers), analysis will produce predicted least square (LS) means differences instead of mean difference. These represent the predicted difference between the observed mean and a mean based on a linear model such as a 2-way ANOVA with multiple comparisons analysis. The 95% confidence interval (CI) represents the range where difference between the two means will fall 95% of the time based on chance. If the actual value is outside this range it is deemed significant with P-value ≤ 0.05. Key: α = 0.05 ∗P <0.05, ∗∗P < 0.01, ∗∗∗P < 0.001, and ∗∗∗∗P < 0.0001, n.s. is non-significant.

## Limitations

This protocol is meant to help establish a drug-based selection system for fly genetics in any lab as an alternative to screen-based genetics using visual markers (Steller and Pirrotta, 1985; Venken and Bellen, 2007). The only limitation is the need to perform titration or survival curves for each drug to establish an effective selection and/or counterselection concentration (ESC/ECC) in a given genetic background due to natural variation in drug resistance/sensitivity. Once established however, selection and/or counterselection genetic strategies can be readily employed. If multiple fly stocks, of differing backgrounds, are to be used in an experiment, it is recommended but not required to establish the required ESC(s) and/or ECC(s) for each stock. If ESC/ECC(s) are not established for all strains in a cross, we recommend simply setting up multiple crosses using the known drug concentration as well as vials drugged with slightly more and/or slightly less drug as a way of roughly estimating the ESC/ECC of the resulting progeny.

A minor limitation of this protocol is that the resulting transgenic flies may not be physically distinct from wild type animals as drug-based selection/counterselection does not rely on physical markers, requiring molecular analysis to confirm successful transgenesis. This can be remedied by the addition of a physical marker into the design of a selection marker containing transgenic construct for simple, visual confirmation of accurate drug selection/counterselection (Kandul et al., 2020; Matinyan et al., 2021; Steller and Pirrotta, 1985).

It is important to note that adult flies remain relatively unaffected by drug treatment (Matinyan et al., 2021; Steller and Pirrotta, 1985). Though this is helpful in terms of allowing non-resistant animals to survive on and lay eggs in drugged food, it does limit drug-based selection/counterselection to developing larvae. However, for tractable genetic manipulations, selection or counterselection is ideal during larval development, removing all unwanted genotypes from the eclosing progeny.

Finally, drug exposure does slow down larval development (Matinyan et al., 2021; Steller and Pirrotta, 1985). Larval development is retarded by about a week on drugged food versus vehicle control at 25°C. The slowdown occurs regardless of genetic background or marker expression. However, the additional time is more than balanced by time saved by not having to screen progeny daily as they eclose. Moreover, once the desired transgenic is generated, animals no longer need to be raised on drugged food. We have not observed any differences in developmental timing or health between wild type and drug marker expressing fly stocks, when raised on regular food.

## Troubleshooting

### Problem 1

Difficulty in dispensing precise amount of food per vial (see step 14)

### Potential solution

While a reasonably cheap food dispensing tool to fill an entire flat of one hundred vials at once is commercially available, known as the Droso-filler, we recommend against using it. Although this tool allows for quick dispensing of food, we find that the resulting volume of food in the vials to be highly variable between vials in the same tray, and from tray to tray. Accurate drugging of the food requires precise dispensing of food into each vial to reduce variability between replicates and ensure accurate results. While the most high-tech solution would be the use of a calibrated, piston-driven pump, there are a number of alternatives to precise dispension that we mention in this protocol. Though time consuming, individually measuring out food into each vial and comparing to a reference vial with known amount inside is necessary if no other alternative is available. Though we have not tried it ourselves, it is likely that a catheter tip syringe, with the tip cut to increase the size of the intake, may allow for precise food dispensing as well.

### Problem 2

Low number of recovered animals but high number of pupal cases (see step 30)

### Potential solution

On occasion, due to high ambient humidity or food quality, a low number of adults flies will be recovered from a vial despite a high number of empty pupal cases on the sides of the vial. In such cases it is likely that many of the adults have fallen into food, making an accurate count of surviving animals difficult. A solution is to instead count the empty pupal cases as a substitute for actual adult flies. The difference between empty and full pupae can be easily seen if the vial is placed in front of a light source as empty pupal cases will be transparent.

### Problem 3

High variability between replicates per concentration (see step 31)

### Potential solution

We recommend always using younger animals for determining effective selection or counterselection concentrations as older animals produce fewer offspring which may introduce high variability when counting surviving flies at the end of the experiment. If running the experiment at 25°C we also recommend acclimating the flies to the elevated temperature by placing them into the incubator 1–2 days prior to setting up the crosses. Increasing the number of vials per concentration or the number of flies per vial is another potential solution though the resulting experiments will require greater numbers of animals.

### Problem 4

Two or fewer fertile replicate crosses for one or more drug concentrations (see quantification and statistical analysis)

### Potential solution

Though unlikely if using flies 3 weeks or younger and acclimated to experimental conditions, on occasion a titration curve may yield only two fertile crosses for a particular drug concentration for a given strain. We always recommend having at least 3 replicates per drug concentration per strain for robust statistical analysis of the results. If the affected concentration is at or near the suspected effective selection/counterselection concentration (ESC/ECC), we recommend redoing the whole titration curve to ensure an accurate result. However, if the ESC/ECC can be determined without including this concentration point, it may not be necessary to repeat the experiment.

### Problem 5

Significant difference in resistant (selection) or wild-type (counterselection) survival at the putative ESC/ECC versus vehicle control (see quantification and statistical analysis)

### Potential solution

It may be possible that after statistical analysis of a user’s survival curve, that at the drug concentration where all non-resistant animals (if determining an effective selection concentration) or where all sensitized animals (if determining an effective counterselection concentration) are totally non-viable, there is a significant difference in the survival of either the drug resistant or wild-type strain versus vehicle control. In this case, it is likely that the true ESC/ECC lies in between this point and the preceding one. Determination of the true ESC/ECC will require a second, smaller drug titration curve focusing on concentrations inbetween these two points. We recommend performing this second, finer drug titration curve.

## Resource availability

### Lead contact

Further information and requests for resources and reagents should be directed to and will be fulfilled by the lead contact, Koen JT Venken (koen.j.t.venken@gmail.com).

### Materials availability

This study did not generate new unique reagents.

## Data Availability

This study did not generate/analyze any data sets/code*.*
